# Challenges and Opportunities in P450 Research on the Eye

**DOI:** 10.1124/dmd.122.001072

**Published:** 2023-10

**Authors:** Irina A. Pikuleva

**Affiliations:** Department of Ophthalmology and Visual Sciences, Case Western Reserve University, Cleveland, Ohio

## Abstract

Of the 57 cytochrome P450 enzymes found in humans, at least 30 have ocular tissues as an expression site. Yet knowledge of the roles of these P450s in the eye is limited, in part because only very few P450 laboratories expanded their research interests to studies of the eye. Hence the goal of this review is to bring attention of the P450 community to the eye and encourage more ocular studies. This review is also intended to be educational for eye researchers and encourage their collaborations with P450 experts. The review starts with a description of the eye, a fascinating sensory organ, and is followed by sections on ocular P450 localizations, specifics of drug delivery to the eye, and individual P450s, which are grouped and presented based on their substrate preferences. In sections describing individual P450s, available eye-relevant information is summarized and concluded by the suggestions on the opportunities in ocular studies of the discussed enzymes. Potential challenges are addressed as well. The conclusion section outlines several practical suggestions on how to initiate eye-related research.

**SIGNIFICANCE STATEMENT:**

This review focuses on the cytochrome P450 enzymes in the eye to encourage their ocular investigations and collaborations between P450 and eye researchers.

## Introduction

**Fig. 1.**
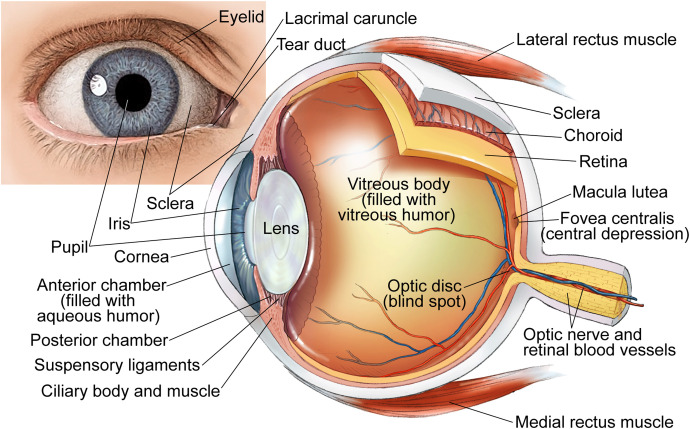
Human eye anatomy. Each eyeball is an oblate spheroid with the mean perimeter of 75 mm and the diameters varying from 24 mm (anterio-posterio) to 23.5 mm (horizontal) and 23 mm (vertical diameter). The average eyeball weighs 7 g and has a volume of 6.5 ml. The eyeball has two segments, anterior (or front) and posterior (or back), which are fused in the middle and communicate through the pupil. The anterior segment includes (from anterior to posterior): cornea, anterior chamber (filled with ∼0.25 ml of the aqueous humor), iris-ciliary body, and lens. The posterior chamber is formed by the vitreous humor (a gel-like material, which fills the space behind the lens), retina, choroid, and optic disc. The eye has three coats or tunics: outer (fibrous), middle (vascular), and inner (nervous). The fibrous coat protects intraocular content and is comprised of the transparent anterior part (cornea) and the opaque posterior part (sclera). The vascular coat supplies nutrition to the various structures of the eyeball and is composed of the iris-ciliary body in the anterior segment and choroid in the posterior segment. The nervous coat (retina) is a sensory tissue in the posterior segment, which receives visual stimuli and initiates the visual process by relaying visual sensations to the to the optic nerve and then to the brain (occipital cortex) (Khurana et al., 2015). This figure was licensed from Carlson Stock Art and is used with permission.

What people see is responsible for approximately 80% of what they learn as well as what they remember (Peterson, 2019). Currently, about 36 million people around the world are blind, another 216.6 million have moderate to severe visual impairment, and 188.5 million have mild visual impairment (Bourne et al., 2017). Blindness is the most feared condition in the US adult population, which ranks losing eyesight as potentially having the greatest effect on their day-to-day life than loss of limb, memory, hearing, or speech (Scott et al., 2016). Yet the role of different cytochrome P450 enzymes (P450s or CYPs) in the structure and function of the eye under normal and pathologic conditions is not yet well understood, in part because only very few P450 laboratories have expanded their research interests to studies of the eye. Other reasons include the complexity of the eye (Fig. 1), the need to learn additional eye-specific in vivo and in vitro characterizations, the cost and difficulty of acquisition of fresh human eyes/eye tissues through eye banks, and the very small size of the eyes of a mouse, the most common laboratory species, thus requiring a large animal colony.

Human eye, the second most powerful and complex organ in the human body after the brain, is composed of >2 million operational parts, including >107 million cells (Peterson, 2019). Hence, studies of P450s in the eye are always a challenge for non-eye researchers. Nevertheless, they are possible, as exemplified by the author of this article who started as a biochemist conducting structure and functional studies on purified cholesterol-metabolizing P450s in vitro and then extended her research expertise to in vivo investigations of cholesterol-metabolizing P450s in the brain and retina.

It has been some time since a publication of the excellent reviews on the P450s in eye (Duvvuri et al., 2003, 2004; Choudhary et al., 2006; Nakano et al., 2014). Therefore, this review provides an updated summary of the P450-releated research in the eye and indicates existing opportunities and challenges in ocular studies of P450s. Perhaps not all the opportunities are mentioned, only several, which reflect the expertise of this article author.

### Expression of Different P450s in the Eye

Currently, cytochromes P450 are represented by more than 300,000 members found in all the biologic phyla so far examined (Nelson, 2018). These enzymes are typically monooxygenases, i.e., they activate molecular oxygen and incorporate one of its atoms in the substrate while reducing the other atom to water. Carbon hydroxylation is a very common P450 reaction. Other common reactions include heteroatom oxygenation, heteroatom release (dealkylation), and epoxidation. The less common reactions are reductions, desaturation, ester cleavage, and others (Guengerich, 2001). A summary of the ocular P450 localizations in different species is provided in Table 1, which, however, does not encompass all P450s that can be present in the eye. This is because several enzymes (e.g., CYP2U1, CYP21A2, and CYP51) have not yet been specifically investigated for ocular location; nevertheless, their ocular importance is suggested by the clinical manifestations of the human gene variants or a phenotype of knockout mice (Xie et al., 2000; Incorvaia et al., 2003; Leonardi et al., 2016; Huang et al., 2019; El Matri et al., 2021; Berry et al., 2022; Gong et al., 2022; Zenteno et al., 2022). Accordingly, it appears that of the 57 P450s found in humans (Nelson et al., 2004), 30 have the eye as an expression site. Ten of these P450s (1A1, 1A2, 2A6, 2B6, 2C9, 2C19, 2D6, 2E1, 3A4, and 3A5) participate in xenobiotic/drug metabolism in extraocular organs; nine P450s (1B1, 11A1, 17A1, 19A1, 21A2, 27A1, 39A1, 46A1, and CYP51) are involved in metabolism of sterols; six P450s are the fatty acid hydroxylases (2C8, 2J2, 2U1, 4A, 4B1, and 4V2), four P450s (26A1, 26B1, 26C1, and 27C1) metabolize vitamin A; and one P450 (4F8) acts on eicosanoids (Guengerich, 2005). The provided P450 grouping (based on the major P450 substrate) is not ideal, as some P450s metabolize more than one major substrate (Guengerich, 2005). Nevertheless, this classification is common and is used in the following sections.

**TABLE 1:** Distribution of P450s in the eye

**P450_species_**	**Cornea**	**Iris**	**Ciliary Body**	**Lens**	**Retina**	**RPE**	**Choroid**	**References**
1A1_r_				RT-PCR				(Nakamura et al., 2005)
1A2 _r_				RT-PCR (nd)				(Nakamura et al., 2005)
1A2_h_	RT-PCR (nd)				RT-PCR (nd)			(Zhang et al., 2008)
1A1/2_m_	IH (ep, end), BNF-ind.	IH (ep), BNF-ind.	IH (ep), BNF-ind.	IH (nd), BNF-ind.	IH (nd), BNF-ind.	IH, BNF-ind.	IH, BNF-ind.	(Zhao and Shichi, 1995)
2A6_h_	RT-PCR				RT-PCR			(Zhang et al., 2008)
2B1_h_	IH (nd)	IH	IH		IH (nd)	IH (nd)		(Doshi et al., 2006)
2B1/2 _r_				RT-PCR				(Nakamura et al., 2005)
2B6_h_	RT-PCR (nd)				RT-PCR (nd)			(Zhang et al., 2008)
2C_m_	IH		IH	IH	IH (GCL, INL, ONL)			(Tsao et al., 2001; Shao et al., 2014)
2C8_h_	RT-PCR	RT-PCR (nd)			RT-PCR			(Zhang et al., 2008)
2C9_h_	RT-PCR	RT-PCR (nd)			RT-PCR (nd)			(Zhang et al., 2008)
2C11_r_				RT-PCR				(Nakamura et al., 2005)
2C19_h_	RT-PCR	RT-PCR (nd)			RT-PCR (nd)			(Zhang et al., 2008)
2D6_h_	RT-PCR							(Zhang et al., 2008)
2D6_p_	IH							(Kölln and Reichl, 2016)
2E1_h_						IH		(Martinez-Gil et al., 2015)
2E1_r_				RT-PCR				(Nakamura et al., 2005)
2J_m_	IH, whole eye homogenate							(Xie et al., 2000)
3A1 _r_				RT-PCR				(Nakamura et al., 2005)
3A2 _r_				RT-PCR (nd)				(Nakamura et al., 2005)
3A4 _h_					RT-PCR (nd)			(Zhang et al., 2008)
3A5_h_	RT-PCR	RT-PCR (nd)			RT-PCR (nd)			(Zhang et al., 2008)
4A1_r_				RT-PCR (nd)				(Nakamura et al., 2005)
4A_m_	IH (ep, end)		IH (ep)		IH (GCL, INL, ONL)	IH		(Zhao et al., 1996)
4B_r_	RT-PCR (ep), CL-injury							(Mastyugin et al., 1999)
4F8_h_	IH (ep)							(Stark et al., 2003)
4V2_h_	IH (ep)				IH (GCL, OPL)	IH	IH	(Nakano et al., 2012)
11A1_h_					IH (GCL, INL, ONL), GC-MS	IH, GC-MS		(Mast et al., 2011; Zheng et al., 2012)
11A1_r_					IH (GCL, INL), GC-MS			(Guarneri et al., 1994; Cascio et al., 2007)
11A1_ham_					IH (GCL, INL)			(Jaliffa et al., 2005)
17A1_r_					IH (GCL, INL, OPL, ONL)			(Cascio et al., 2007)
19A1_m_					IH (all retinal layers)			(Iglesias-Osma et al., 2022)
19A1_h_	RT-PCR							(Schirra et al., 2006)
19A1_r_					IH (INL, OPL, ONL, IS)			(Cascio et al., 2007)
26A1*_Xl_*	RT-PCR, IH			RT-PCR				(Thomas and Henry, 2014)
26B1*_Xl_*	RT-PCR, IH			RT-PCR				(Thomas and Henry, 2014)
26C1*_Xl_*				RT-PCR				(Thomas and Henry, 2014)
27A1_monk_					IH (GCL, MC, IS)	IH	IH	(Lee et al., 2006)
27A1_h_					IH (GCL, INL, MC, IS), MS, GC-MS	RT-PCR, IH, MS		(Heo et al., 2011; Liao et al., 2011; Mast et al., 2011; Wang et al., 2012; Zheng et al., 2012)
27C1_z, bg_						RT-PCR, IH, TH-treated		(Enright et al., 2015)
39A1_cow_			IH					(Ikeda et al., 2003)
46A1_h_					IH (GCL, IS), MS, GC-MS	IH, MS, GC-MS		(Liao et al., 2011; Mast et al., 2011; Wang et al., 2012; Zheng et al., 2012)
46A1_m_					IH (GCL, INL)	IH		(Ramirez et al., 2008)
46A1 _r_					IH (GCL, INL)			(Bretillon et al., 2007)
46A1_cow_			GC-MS		RT-PCR, GC-MS	RT-PCR, GC-MS		(Bretillon et al., 2007)

Only studies on tissue samples were considered, and data on P450 expression in cell cultures are not included as these data may be confounded by cell isolation and maintenance. Most of the described localizations were conducted by RT-PCR and/or immunohistochemistry to assess the gene and protein expression, respectively. Yet, in some cases, mass spectrometry was used to quantify the P450 protein levels and the presence of the P450-specific metabolites. Since RT-PCR, immunohistochemistry, and mass spectrometry have their limitations, and tissue samples were from different species, there may be a discrepancy between the data obtained by different approaches. Use of different P450 inducers may also contribute to data discrepancy.

BNF-ind, beta-naphthoflavone-induced; CL, the contact lens-induced hypoxic injury; end, endothelial; ep, epithelial; h, human; ham, hamster; GCL, the ganglion cell layer; GC-MS, gas chromatography-mass spectrometry; m, mouse; monk, monkey; MC, Muller cells; MS, mass spectrometry; IH, immunohistochemistry; INL, the inner nuclear cell layer; IS, the photoreceptor inner segments; nd, not detectable; ONL, the outer nuclear layer; OPL, the outer plexiform layer; p, pig; r, rat; RPE, retinal pigment epithelium; RT-PCR, reverse-transcription polymerase chain reaction; TH, thyroid hormone; Xl, Xenopus laevis; z, zebrafish.

### Drug Delivery to the Eye

**Fig. 2.**
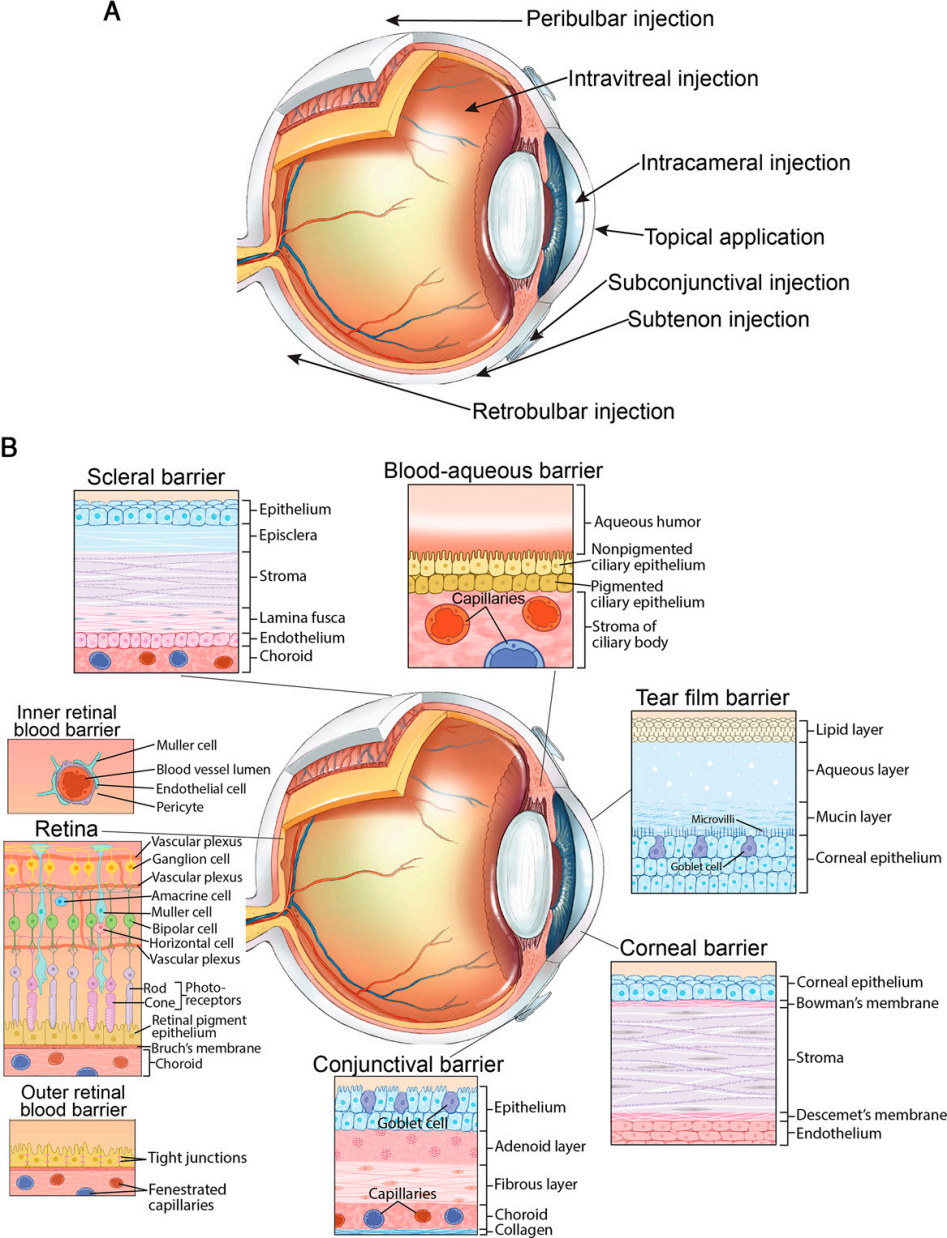
Routes and anatomic barriers for ocular drug delivery. (A) Unique routes of drug delivery to the eye include topical applications, periocular injections, and intraocular injections. Topical applications are administered in the form of eyedrops, ointments, gels, or ocuserts (membranes) or via soft contact lenses. Periocular injections include subconjunctival, subtenon, retrobulbar, and peribulbar injections. Intraocular injections are represented by intracameral injections (into the anterior chamber) and intravitreal injections (into the vitreous cavity). (Khurana et al., 2015). (B) The tear film, cornea, conjunctiva, sclera, BAB, and BRB represent the anatomic barriers for drug delivery to the eye. The tear film is composed of the three layers, including the external lipid layer, which covers the water layer and mucin layer. The lipid layer of the tear film limits access of aqueous formulations to the cornea. In addition, the aqueous phase under the lipid layer is rich in enzymes, proteins, and mucins that can inactivate drugs by protein binding or enzymatic degradation, thus reducing their bioavailability. A high turnover rate of the tear film dilutes topical drugs, and the blink reflux washes them away within 15 to 30 seconds after instillation (Arturo et al., 2019; Agarwal et al., 2021). The cornea also has three major layers: epithelium, stroma, and endothelium. The corneal epithelium acts as a physical barrier to hydrophilic drugs, and the corneal stroma is a barrier to lipophilic drugs (Gaudana et al., 2010; Janagam et al., 2017). Overall, the cornea favors the absorption of lipophilic over hydrophilic drugs (Farkouh et al., 2016). The conjunctiva is a thin mucous membrane consisting of the conjunctival epithelium and an underlying vascularized connective tissue. The conjunctival epithelium is relatively leaky and hydrophilic, with intercellular spaces approximately 230-fold larger than those in the cornea, rendering it permeable even to large biomolecules, such as proteins and peptides. The conjunctiva is more permeable than the cornea for hydrophilic drugs and acts as a dynamic barrier to hydrophobic drugs (Dhahir et al., 2021). The sclera (the white of the eye) consists of collagen and elastin chains that create a fiber matrix, where the pore diameter and intracellular spaces determine the drug flow with smaller molecules having a better permeability (Arturo et al., 2019). Positively charged molecules have poor permeability through the sclera, presumably due to their binding to the negatively charged proteoglycan matrix (Gaudana et al., 2010). Both the sclera and conjunctiva are richly perfused with blood vessels, hence a large fraction of drugs absorbed via these routes may be lost to systemic circulation (Agarwal et al., 2021). As compared with the corneal route, the conjunctiva and sclera are considered to be the minor pathways for drug delivery (Dosmar et al., 2022). The blood-ocular barriers, BAB and BRB, restrict drug entry from the systemic circulation. The BAB is formed by the capillary endothelium in the iris and the ciliary epithelium, which both contain tight junctions. The BRB is formed by the retinal pigment epithelium (outer BRB) and the endothelial membrane of the retinal blood vessels (inner BRB), which also contain tight junctions (Janagam et al., 2017). The BAB is relatively inefficient as compared with the BRB, and small molecules can cross it by permeation through fenestrated capillaries in the ciliary processes (Janagam et al., 2017). The BRB limits drug passage more efficiently than BAB with small lipophilic molecules crossing the BRB comparatively better than hydrophilic or large molecular weight compounds (Ako-Adounvo and Karla, 2018). All panels for this figure were licensed from Carlson Stock Art and are used with permission. BAB, blood-aqueous barrier; BRB, blood-retinal barrier.

The unique structure of the eye and its partial exposure to the external environment determine distinctive routes of drug delivery to the eye, which include topical applications, periocular injections, and intraocular injections (Fig. 2A). In addition, drugs can enter the eye from the systemic circulation after oral intake, subcutaneous, intramuscular, or intravenous injections (Khurana et al., 2015). Different anatomic barriers restrict ocular drug delivery (Fig. 2B). The tear film, cornea, conjunctiva, and sclera limit drug penetration to the anterior segment, whereas the blood-ocular barriers (blood-aqueous barrier and blood-retinal barrier) restrict drug access to the posterior segment. Topical and systemic administrations are the least invasive routes for delivering medications to the eye. The former are also the most commonly employed mode of ocular drug administration with >90% of the marketed ophthalmic formulations being in the form of eye drops (Gaudana et al., 2009). Topical drugs largely penetrate through the cornea and, by avoiding first-pass hepatic metabolism, reduce the need for higher dosing associated with oral administration. However, only 1% to 7% of the topically administered drugs can reach the aqueous humor due to the tear film, corneal and conjunctival barriers, as well as lacrimation (tear secretion), tear dilution, reflex blinking, and nasolacrimal drainage, the latter accounting for 80% to 90% of drug elimination (Janagam et al., 2017; Arturo et al., 2019). In the case of a systemic administration, only 1% to 2% of the drug reaches the vitreous cavity (Gaudana et al., 2009).

Periocular injections (subconjunctival, subtenon, retrobulbar, and peribulbar) represent a deposition of therapeutic agents into the areas adjacent to or surrounding the eye. Periocular injections are used less frequently than topical instillations and are more invasive. Nevertheless, they are still relatively safe for delivering drugs that cannot penetrate the cornea but can easily pass through the sclera. Periocular injections take advantage of the large sclera area for drug absorption (95% of the surface of the eyeball) and are partly used to overcome the inefficiency of topical and systemic dosing to deliver therapeutic drug concentrations to the posterior segment of the eyes. Periocular injections are commonly used to administer local anesthetics and corticosteroids (Ako-Adounvo and Karla, 2018) with subtenon injections resulting in the highest and more sustained vitreous concentration of drug molecules compared to other periocular injections (Arturo et al., 2019).

Intraocular injections and implants are used only in certain cases to deliver the maximum drug concentration to the target tissue. Intraocular injections include intracameral injections (into the anterior chamber) and intravitreal injections (into the vitreous cavity). Intravitreal drug implants are inserted in the vitreous cavity for sustained and slow release. Intravitreal injections are an invasive route of drug administration with adverse events including endophthalmitis, retinal detachment, intraocular hemorrhage, and particulate contamination. Additionally, the need for frequent administration results in a significant treatment burden to patients, a high-volume burden on providers, and an increased cumulative risk of adverse events. Nevertheless, because of the therapeutic benefits, intravitreal injections are the preferred route for ocular delivery to the retina, specifically of the antivascular endothelial growth factor agents, corticosteroids, and some of the antibiotics (Ako-Adounvo and Karla, 2018; Arturo et al., 2019).

### Drug-Metabolizing P450s

At least 10 human drug-metabolizing P450s (1A1, 1A2, 2A6, 2B6, 2C9, 2C19, 2D6, 2E1, 3A4, and 3A5) seem to be expressed in the anterior part of the eye (Table 1), which is affected by administration of ocular topical mediations, the most common marketed ophthalmic formulations (Gaudana et al., 2009). Notably, some of these topical medications (substrates, inducers, or inhibitors of P450s) are known to elicit both ocular and systemic effects (Syed et al., 2021). For example, among the drugs for glaucoma treatment, a major cause of legal blindness (Quigley and Broman, 2006), these are timolol metabolized by CYP2D6; betaxolol, a substrate for CYP1A2 and CYP2D6; dorzolamide eliminated by CYP2B1, CYP2C9, CYP2E1, and CYP3A2; and pilocarpine, which inhibits CYP2A6, CYP2A13, and CYP2E1 (Kimonen et al., 1995). Particular caution is recommended when ophthalmic timolol or betaxolol are coadministered with paroxetine or other strong CYP2D6 inhibitors (Farkouh et al., 2016; Vaajanen and Vapaatalo, 2017).

Some of the systemically administered drugs also elicit serious ocular effects in the anterior and/or posterior parts of the eye (Syed et al., 2021). These include the antimalarial hydroxychloroquine and chloroquine (inhibit CYP2D6 and are metabolized by CYP2C8 and CYP3A4/5) (Rendic and Guengerich, 2020); the antiarrhythmic amiodarone (inhibits CYP1A2, CYP2C9, CYP2D6, and CYP3A4 and is metabolized by CYP3A4) (McDonald et al., 2015); the antiarrhythmic propranolol (metabolized by CYP1A2 and CYP2D6) (Johnson et al., 2000); the antiarrhythmic quinidine (inhibits CYP2D6 and is metabolized by CYP3A4) (Guengerich et al., 1986; Branch et al., 2000); the antidepressant fluoxetine (both an inhibitor and a substrate of CYP2D6) (Lynch and Price, 2007); the antidepressant fluvoxamine (inhibits CYP1A2) (Brøsen et al., 1993); cholesterol-lowering simvastatin, lovastatin, and atorvastatin (metabolized by CYP3A4) (Bellosta et al., 2004); the antituberculosis ethambutol (inhibits CYP1A2 and CYP2E1) (Lee et al., 2014); and the anticancer tamoxifen (metabolized by CYP2D6, CYP3A4, CYP2B6, and CYP2C19) (Singh et al., 2011).

A list of drugs with ocular side effects is much more extensive than that indicated here. Therefore, readers are referred to several review and experimental articles to learn more either about these drugs or specific manifestations of their ocular toxicity (Shichi and Nebert, 1982; Siu et al., 2008; Farkouh et al., 2016; Novack and Robin, 2016; Vaajanen and Vapaatalo, 2017; Morawski et al., 2020; Syed et al., 2021; Souza Monteiro de Araújo et al., 2022). The provided examples are meant to illustrate the importance of studies in the eye of drug pharmacokinetics and pharmacodynamics as well as drug–drug interactions and genetic differences in ocular drug metabolism. These areas of research represent both a challenge and an opportunity for the P450 community. A challenge is in the unique anatomy and complexity of the eye and the difficulty of obtaining samples of ocular tissues for drug quantifications. An opportunity is the multiple routes of drug administration to the eye, which can be used to improve ocular safety of the marketed drugs.

### Sterol-Metabolizing P450s

#### CYP1B1

Of the nine sterol-metabolizing P450s that seem to be present in the eye (1B1, 11A1, 17A1, 19A1, 21A2, 27A1, 39A1, 46A1, and CYP51), CYP1B1 stands apart from the remaining eight enzymes (the classic endobiotic-metabolizing P450s) because it can use both endogenous and exogenous substrates. The former include 17*β*-estradiol, arachidonic acid, vitamin A, and melatonin (Jansson et al., 2001; Choudhary et al., 2004; Ma et al., 2005; Choudhary et al., 2006, 2008; Nakano et al., 2014). The latter are exemplified by procarcinogens, namely, polycyclic aromatic hydrocarbons, heterocyclic amines, and aromatic amines (Guengerich, 2005)]. In addition, CYP1B1 is perhaps one of the most well-known P450s among eye researchers as, in 1997, *CYP1B1* was identified as a causative gene for primary congenital glaucoma (PCG), the major glaucoma type in the pediatric population. Later, *CYP1B1* was found to be a modifier gene for primary open-angle glaucoma (POAG), the major glaucoma type in the adult population (Stoilov et al., 1997; Quigley and Broman, 2006; Vasiliou and Gonzalez, 2008; Aponte et al., 2010). Now more than 150 *CYP1B1* mutations have been found in subjects with PCG (Rauf et al., 2016), and, of them, approximately two-thirds are missense mutations (Li et al., 2011). Of the latter, 23 of the most frequent *CYP1B1* mutations were studied for the effect on enzyme activity in a cell-based system, and most variants were established to have reduced metabolism of 17*β*-estradiol and absent or increased metabolism of retinol (Banerjee et al., 2016). Intriguingly, the *CYP1B1* mutation penetrance is variable in the human population (Bejjani et al., 1998, 2000), and it is currently unknown why.

Strong CYP1B1 expression in the fetal human and mouse eyes suggested the enzyme role in ocular development and function (Hakkola et al., 1997; Stoilov et al., 1997). Hence, CYP1B1 was proposed to mediate the metabolism of endogenous and exogenous compounds that are important for eye development (Stoilov et al., 1997; Choudhary et al., 2006; Vasiliou and Gonzalez, 2008). Also, CYP1B1 was suggested to coordinate the expression of some important genes relevant to the anterior chamber formation (Stoilov, 2001; Stoilov et al., 2001). These proposed roles of CYP1B1 were supported by the characterizations of *Cyp1b1^−/−^* mice, which were found to have developmental abnormalities similar to human PCG (Libby et al., 2003; Zhao et al., 2013; Teixeira et al., 2015). Recent studies also pointed to the CYP1B1 roles in the regulation of ocular iron homeostasis, oxidative stress, expression of the peroxisome proliferator-activated receptor (PPAR) *γ* target genes, and retinal neovascularization (Falero-Perez et al., 2018, 2019a, b; Song et al., 2022). Nevertheless, despite all these investigations, the key processes affected by CYP1B1 activity in the anterior and posterior parts of the eye and precise mechanisms by which CYP1B1 mutations underlie the development of PCG are currently not well understood. Accordingly, important areas in CYP1B1 research include uncovering CYP1B1 ocular significance and identification of both endogenous and exogenous CYP1B1 substrates that pertain to the PCG development. Both areas represent an opportunity for P450 investigators and simultaneously are a challenge as highlighted by the fact that these areas have remained a priority in the CYP1B1 ocular research for 25 years.

#### Steroidogenic P450s

**Fig. 3.**
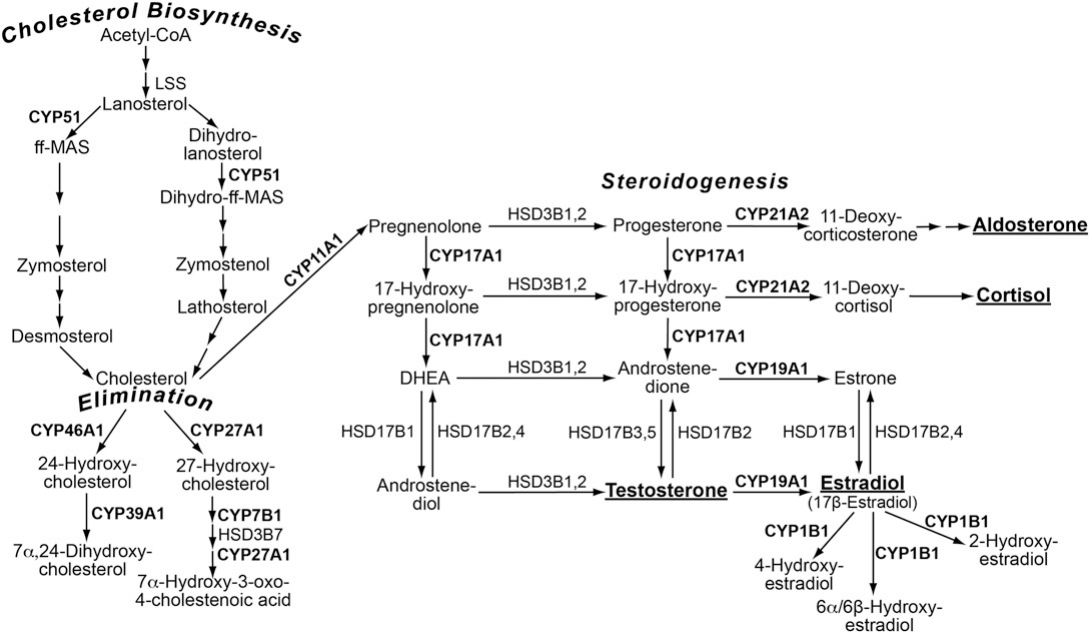
Sterol-metabolizing P450s expressed or implicated to be expressed in the eye. The P450 (in bold) grouping is by the biologic process (in bold italics). Not all the steps in the indicated three biologic processes are shown—only those involving the discussed P450 enzymes. Two or three arrows indicate multiple enzymatic reactions. The primary steroid hormones are in bold and underlined. See text for details.

CYPs 11A1, 17A1, 19A1, and 21A2 represent steroidogenic P450s (Fig. 3) as they catalyze the key steps in the production of different steroid hormones, namely glucocorticoids, mineralocorticoids, and sex hormones (androgens and estrogens) (Miller and Auchus, 2011). CYP11A1 converts cholesterol to pregnenolone, the precursor of all steroid hormones. Pregnenolone and its metabolite progesterone then could be hydroxylated by CYP17A1 to yield 17*α*-hydroxypregnenolone and 17*α*-hydroxyprogesterone, respectively, steroids that can be further metabolized by CYP17A1 to the androgens dehydroepiandrosterone and androstenedione, respectively. Dehydroepiandrosterone and androstenedione are precursors of the primary sex hormone testosterone, which can be converted to another primary sex hormone estradiol via the action of CYP19A1 that catalyzes the aromatization of androstenedione and testosterone. 17*α*-Hydroxyprogesterone produced by CYP17A1 can also serve as a substrate for CYP21A2 to ultimately generate the primary glucocorticoid cortisol. Progesterone, which is formed from pregnenolone, is another substrate for CYP21A2 and a precursor for aldosterone, the primary mineralocorticoid.

The indicated P450-mediated reactions outline the principal pathways of steroidogenesis. Yet which of these pathways is operative and which types of steroid hormones are predominantly produced depends on a steroidogenic tissue and the cell type (Miller and Auchus, 2011). For example, corticosteroids (mineralocorticoids and glucocorticoids) are typically synthesized by the adrenal glands (in the zona glomerulosa and zona fasciculata, respectively), whereas sex hormones are mainly made in the gonads or placenta with some contribution from the zona reticularis of the adrenals, which produces androgens (Miller and Auchus, 2011). In addition, small amounts of steroid hormones are synthesized in the brain and constitute a local regulatory mechanism for different brain processes (Corpéchot et al., 1981; Baulieu, 1997).

Steroid hormones exert a significant influence on the health and well-being of the eye as receptors for steroid hormones are found in ocular structures in both the anterior (the lacrimal gland, meibomian gland, conjunctiva, goblet cells, cornea, anterior chamber, iris, ciliary body, and lens) and posterior parts of the eye (the vitreous humor and retina) (Sullivan, 2004). Accordingly, steroid hormones influence multiple structural and functional aspects of the eye. These include tissue morphology, epithelial cell dynamics (e.g., proliferation, maturation, transport, and secretion), aqueous tear output, lipid production, mucous secretion, tear film stability, corneal properties (e.g., thickness, curvature, sensitivity, and wetting time), goblet cell density, aqueous humor circulation and outflow, intraocular pressure, lens epithelial cell density, lens permeability, retinal thickness, optic cup area, as well as visual coordination and acuity (Sullivan, 2004; McKay et al., 2022). In addition, steroid hormones (mainly sex hormones) have been linked to the development, progression, and/or treatment of many ocular conditions, including dry eye syndromes, contact lens intolerance, allergic and vernal keratoconjunctivitis, allergic conjunctivitis, corneal angiogenesis, wound healing, transplant rejection, refractive errors, keratitis, myopia, keratoconus, cataracts, glaucoma, amblyopia, photophobia, optic neuritis, papilloedema, age-related macular degeneration (AMD), retinal vascular occlusion, retinal neuron apoptosis, and diabetic retinopathy (Sullivan, 2004).

However, it not clear whether hormonal ocular effects are solely due to the action of the hormones delivered from the systemic circulation or there is also some ocular steroidogenesis either from cholesterol or blood-borne steroid precursors. Several lines of evidence support the notion of local steroidogenesis in different oculars structures: first, detection of steroidogenic P450s in these structures (Table 1); second, capacity of some of these structures (e.g., the retina and cornea) to synthesize steroid hormones either ex vivo or in cultures of cells from these structures (Guarneri et al., 1994, 2003; Susarla et al., 2014; Cascio et al., 2015); and third, ocular manifestations of a steroidogenic P450 deficiency. The latter is exemplified by CYP21A2, which is necessary for mineralocorticoid and glucocorticoid production in the adrenal glands and whose deficiency leads to most cases of congenital adrenal hyperplasia (Miller and Auchus, 2011). Recently several pathogenic variants of CYP21A2 have been linked to autosomal dominant congenital cataracts (Berry et al., 2022), and earlier CYP21A2 was detected in cultures of human lens epithelial cells (Zhang et al., 2013). Collectively, these findings suggested that CYP21A2 could be important for biosynthesis of aldosterone and cortisol in the lens. In addition, CYP21A2 deficiency could be associated with keratoconus, a condition in which the cornea assumes a conical shape due to thinning of the corneal stroma (Incorvaia et al., 2003). Yet there are no reports on CYP21A2 expression in the cornea; rather, primary human corneal epithelial cells were shown to synthesize cortisol from cortisone (Susarla et al., 2014). Thus, establishing the major types and sources of steroid hormones in different ocular structures is an opportunity for P450 researchers, which will help to better control the physiology of ocular tissues and treat various disorders of the eye.

#### Cholesterol-Metabolizing P450s

In the eye, these include CYP11A1 (discussed in the previous section) as well as CYP27A1 and CYP46A1 that use cholesterol as the endogenous substrate along with CYP39A1 that acts on the cholesterol metabolite produced by CYP46A1 (Fig. 3). CYP27A1 sequentially hydroxylates cholesterol at C27 to yield 27-oxygenated sterols (27-hydroxycholesterol, 5-cholestenoic acid, and 7*α*-hydroxy-3-oxo-4-cholestenoic acid), whereas CYP46A1 hydroxylates cholesterol at C24 to generate 24-hydroxycholesterol (Wikvall, 1984; Pikuleva et al., 1998; Lund et al., 1999; Meaney et al., 2007). 24-Hydroxycholesterol could then be hydroxylated by CYP39A1 at C7 to produce 7*α*, 24-dihydroxycholesterol (Li-Hawkins et al., 2000).

So far, CYP27A1 and CYP46A1 have been mainly studied in the retina. This is because cholesterol is the major component of drusen and subretinal drusenoid deposits, two extracellular lesions and notable hallmarks of AMD (Curcio et al., 2005; Wang et al., 2010; Oak et al., 2014), a major cause of legal blindness in the elderly of industrialized countries (Wong et al., 2014). Also, evidence has linked dysregulation of the chorioretinal cholesterol homeostasis and AMD (Pikuleva and Curcio, 2014). Second, CYP27A1 deficiency in humans leads to cerebrotendinous xanthomatosis (Cali et al., 1991), a lipid storage disorder, which has ocular manifestations, including those in the retina (Koyama et al., 2021). These retinal manifestations are premature retinal senescence with drusen and retinal vessel sclerosis, cholesterol-like deposits along the vascular arcades, retinal pigment epithelium (RPE) defects on fluorescein angiography as well as optic disc pallor and neuritis (Cruysberg et al., 1995; Dotti et al., 2001; Miyamoto et al., 2019; Koyama et al., 2021). Third, CYP46A1 may play a role in glaucoma and diabetic retinopathy (Saadane et al., 2019; Zhang et al., 2021). A polymorphism in *CYP46A1* was linked to increased risk of POAG (Fourgeux et al., 2009). However, this association was not confirmed in a subsequent study (Mossböck et al., 2011). Despite these conflicting reports, data suggest that 24-hydroxycholesterol could be an endogenous neuroprotectant under glaucomatous conditions (Ishikawa et al., 2016, 2018; Zhang et al., 2021). In addition, CYP46A1 could play a protective role in vascular damage in diabetic retinopathy (Saadane et al., 2019).

CYP27A1 and CYP46A1 have been immunolocalized to the retinal layers and cell types in different species (Lee et al., 2006; Bretillon et al., 2007; Ramirez et al., 2008; Zheng et al., 2012). The retinal abundance of these proteins and retinal levels of their metabolites were quantified by mass spectrometry (Liao et al., 2011; Mast et al., 2011; Wang et al., 2012). Retinal phenotype of *Cyp27a1^−/−^*, *Cyp46a1^−/−^*, and *Cyp27a1^−/−^Cyp46a1^−/−^* mice was extensively characterized and found to lead to retinal cholesterol accumulation and chorioretinal vascular abnormalities (Omarova et al., 2012; Saadane et al., 2014, 2019). CYP27A1 was discovered to be post-translationally modified in the AMD-affected human retina by isolevuglandins, arachidonate oxidation products, which diminished the enzyme activity (Charvet et al., 2011, 2013a). Importantly, in mice, pretreatment with pyridoxamine, a B6 vitamer and efficient scavenger of gamma-ketoaldehydes, reduced the levels of retinal isolevuglandin adducts and mitigated the isolevuglandin-associated retinal effects in animals exposed to bright light (Charvet et al., 2013b). CYP46A1 was studied as a pharmacologic target for enzyme inhibition and activation (Fourgeux et al., 2014; El-Darzi et al., 2022), and the latter was found to be beneficial in 5XFAD mice, a model of Alzheimer’s disease. Retinal CYP46A1 activation enhanced retinal cholesterol turnover and reduced more than fivefold retinal frequency of vascular lesions associated with deposits within the RPE and subretinal space (El-Darzi et al., 2022).

Despite extensive studies of CYP27A1 and CYP46A1 in the retina, opportunities still exist for expanding our knowledge of ocular significance of these P450s. For example, we still do not know the quantitative contributions of the CYP27A1- and CYP46A1-mediated retinal cholesterol removal to the total retinal cholesterol output or whether these contributions are similar in different species (e.g., mice, hamsters, and humans). Also, *Cyp27a1^−/−^* mice do not recapitulate all the features of CYP27A1 deficiency in humans and therefore do not develop cerebrotendinous xanthomatosis (Rosen et al., 1998; Dubrac et al., 2005). Accordingly, we likely underestimate ocular roles of CYP27A1 in humans based on studies of *Cyp27a1^−/−^* mice. Another opportunity is to characterize CYP27A1 and CYP46A1 in ocular structures other than the retina where cholesterol is abundant. Indeed, the content of cholesterol in the membranes of human lens is the highest of any known biologic membrane (Li et al., 1985; Cenedella, 1996). Yet little is known how cholesterol homeostasis is maintained in the lens. What is known, however, is that CYP27A1 deficiency in humans leads to juvenile bilateral cataracts (Morgan et al., 1989; Cruysberg et al., 1995; Dotti et al., 2001), and an SNP in *CYP46A1* is associated with senile cataracts (Raza et al., 2017), the most common cause of blindness worldwide (Resnikoff et al., 2004). Thus, an opportunity is to delineate the role of lenticular cholesterol metabolism in juvenile and senile cataract formation. Perhaps the cornea and nonpigmented ciliary body epithelium could be studied as well, because CYP27A1 was found to be expressed in the cell lines of human corneal endothelium and nonpigmented ciliary body epithelium (Alsalem et al., 2014).

#### CYP51

CYP51 is the only P450 involved in the biosynthesis of cholesterol, where it catalyzes lanosterol and dihydrolanosterol 14*α*-demethylation (Fig. 3) (Debeljak et al., 2003; Lepesheva and Waterman, 2004). In the retina, local cholesterol biosynthesis accounts for the majority of retinal cholesterol input (Lin et al., 2016). Lens and cornea can also synthesize cholesterol (Cenedella, 1982; Hitchener and Cenedella, 1985; Cenedella and Fleschner, 1989), and studies show that the origin of cholesterol in the cornea is of importance. The latter is highlighted by corneal opacification in different genetic diseases (e.g., LCAT and APOA1 deficiencies, Tangier disease, and Schnyder corneal dystrophy) due to cholesterol accumulation (Cenedella and Fleschner, 1989; Cogan et al., 1992; Gaynor et al., 1996; Flores et al., 2019). Conversely, in the lens, reduction in cholesterol content due to inherited defects in the enzymes involved in cholesterol biosynthesis (7-dehydrocholesterol reductase or lanosterol synthase) or use of drugs (lovastatin and simvastatin) that inhibit lens cholesterol biosynthesis can be associated with cataracts in both animals and humans (Cenedella, 1996; Mori et al., 2006; Zhao et al., 2015; Widomska and Subczynski, 2019). Remarkably, the CYP51 substrate lanosterol was found to be a key molecule in the prevention of lens protein aggregation and was suggested to represent a novel strategy for cataract prevention and treatment (Mori et al., 2006; Zhao et al., 2015). Thus, an opportunity for P450 researchers is to study the role of CYP51 in the retina, cornea, and lens as CYP51 should be definitely expressed in these ocular structures. However, currently there does not seem to be any published studies on CYP51 in the eye.

#### Fatty Acid-Hydroxylating P450s

**Fig. 4.**
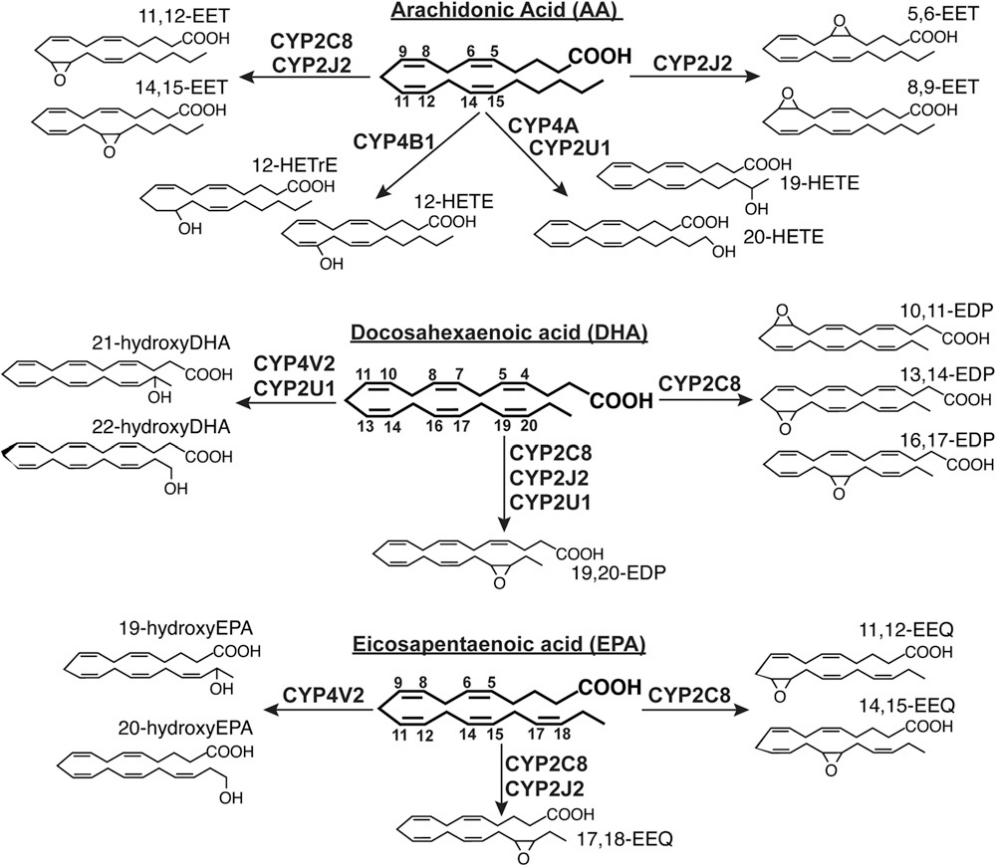
Some of the cytochrome P450 metabolites generated from the long-chain polyunsaturated fatty acids. Not all the P450s (in bold), the long-chain polyunsaturated fatty acids (in bold and underlined), and the products of their metabolism are indicated—only those discussed in the text. The non-P450 enzymes are also not indicated. See text for details. EDP, epoxydocosapentaenoic acid; EEQ, epoxyeicosatetraenoic acid; EET, epoxyeicosatrienoic acid; HETE, hydroxyeicosatetraeonic acid; HETrE, hydroxyeicosatrienoic acid.

Currently, the activities of six fatty acid-hydroxylating P450s (2C8, 2J2, 2U1, 4A, 4B1, and 4V2) seem to be of importance for normal and pathologic processes in the eye. These activities are epoxygenation and/or hydroxylation of long-chain polyunsaturated fatty acids (LPUFAs)—*ω*-6 [arachidonic acid (AA)] and *ω*-3 [docosahexaenoic acid (DHA) and eicosapentaenoic acid (EPA)] (Fig. 4) as well as *ω*/*ω*-1 hydroxylation of short- to medium-chain saturated fatty acids. Many of these activities produce the biologically active metabolites (Fleming, 2014; Ni and Liu, 2021).

Specifically, of the P450s from family 2, CYP2C8 can epoxygenate AA, DHA, and EPA at comparable rates to generate 14,15- and 11,12-epoxyeicosatrienoic acids (EETs) from AA; 19,20-, 16,17-, 13,14-, and 10,11-epoxydocosapentaenoic acid (EDPs) from DHA; and 17,18-, 14,15-, and 11,12-epoxyeicosa-tetraenoic acids (EEQs) from EPA with a minor formation of hydroxylated metabolites (Fer et al., 2008; Arnold et al., 2010). In addition, CYP2C8 can metabolize non-fatty acid substrates, which include all-*trans*-retinol and all-*trans*-retinoic acid and some drugs (e.g., paclitaxel, troglitazone, verapamil, rosiglitazone, cerivastatin, amiodarone, dapsone, and others) (Leo et al., 1989; Rendic, 2002). However, the contribution of CYP2C8 to the metabolism of clinically relevant drugs is not as significant as of CYP2C9 and other major drug-metabolizing P450s (Johnson et al., 2021). CYP2J2 catalyzes the epoxidation of the all four olefin bonds in AA to produce 14,15-, 11,12-, 8,9- and 5,6-EETs (Wu et al., 1996). However, EPA and DHA are much better substrates for CYP2J2 than AA, which mostly undergo epoxidation to 17,18-EEQ and 19,20-EDP, respectively (Fer et al., 2008; Arnold et al., 2010). CY2U1 catalyzes the *ω*/*ω*-1 hydroxylation of both saturated (palmitic and stearic acids but not lauric acid) and unsaturated fatty acids, including AA, EPA, and DHA. 19-Hydroxyeicosatetraeonic acid (HETE), 20-HETE, and either 22- and 21-hydroxyDHAs or 19,20-EDP are some of the identified CY2U1 metabolites (Chuang et al., 2004).

Of the P450s from family 4, the CYP4A isoforms hydroxylate medium-chain saturated fatty acids (e.g., lauric acid) as well as AA at the *ω*/*ω*-1 positions to generate 12- and 11-hydroxylauric acids and 20- and 19-HETEs, respectively (Okita and Okita, 2001). The CYP4A isoforms are the predominant fatty acid *ω*/*ω*-1 hydroxylases in most mammalian tissues (Capdevila et al., 2000). The substrate preferences of CYP4B1 are broader with the typical endogenous substrates being short- to medium-chain saturated fatty acids, which are *ω*-hydroxylated, and also AA, which was proposed to be C12-hydroxylated to yield 12-HETE and 12-hydroxy-5,8,14-eicosatrienoic acid (12-HETrE) (Mastyugin et al., 1999; Baer and Rettie, 2006; Nakano et al., 2014). In addition, CYP4B1 bioactivates a range of xenobiotic protoxins that often exert tissue-specific effects (Baer and Rettie, 2006). CYP4V2 was shown to be a selective *ω*-hydroxylase of the medium-chain saturated fatty acid and also have the *ω*-hydroxylase activity toward DHA and EPA to produce 19-hydroxyEPA, 20-hydroxyEPA, 21-hydroxyDHA, and 22-hydroxyDHA. Overall, CYP4V2 seems to be an efficient *ω*-hydroxylase of both saturated and unsaturated fatty acids (Nakano et al., 2009, 2012).

The DHA and EPA metabolites of CYP2C8 (19,20-EDP and 14,15-EEQ) were found to be proangiogenic and induce retinal neovascularization in mice, counteracting in part the overall antiangiogenic effects of DHA and EPA (Shao et al., 2014). Subsequent studies of CYP2C8-overexpressing mice fed the DHA+EPA-enriched diet and treated with montelukast as selective CYP2C8 inhibitor (Walsky et al., 2005b) showed a significant reduction in the plasma levels of the CYP2C8 products and suppression of pathologic angiogenesis in oxygen-induced retinopathy and laser-induced choroidal neovascularization (CNV) (Gong et al., 2016a). Also, fenofibrate, a PPAR*α* agonist and drug that reduces progression of diabetic retinopathy in type 2 diabetes patients independent of its PPAR*α* effects, suppressed retinal and choroidal neovascularization in mice overexpressing CYP2C8 in endothelial cells and reduced plasma levels of the CYP2C8 metabolite 19,20-EDP. As fenofibrate is a modest CYP2C8 inhibitor (Walsky et al., 2005a), this study suggested that the fenofibrate neovascular effects are mediated via both the PPAR*α* agonist activity and CYP2C inhibition (Gong et al., 2016b). The PPAR*α*-mediated effect on pathologic neovascularization was confirmed in a different study in mouse models that recapitulate some of the features of AMD (Qiu et al., 2017).

Conversely, a different group found that 19,20-EDP and 17,18-EEQ suppressed laser-induced CNV in mice (Yanai et al., 2014) and that animals overexpressing CYP2C8 in endothelial cells and fed the DHA+EPA-enriched diet had markedly attenuated neovascular lesions (Hasegawa et al., 2017). Apparently, the individual effects of 19,20-EDP, 14,15-EET, and 17,18-EEQ on ocular neovascularization need to be investigated to resolve the conflicting data on the CYP2C8 ocular significance. In the meantime, studies in cell culture and rat model of oxygen-induced retinopathy suggested that the AA metabolite 11,12-EET may have a proangiogenic effect in the retina (Capozzi et al., 2014).

As compared with CYP2C8, ocular studies of CYP2J2 are not as extensive but are also conflicting. Endothelium-specific overexpression of CYP2J2 in rats was shown to prevent vascular endothelial senescence and thereby attenuate retinal ganglion cell loss induced by retinal ischemia-reperfusion injury (Huang et al., 2019). In a different study, the laser-induced CNV was exacerbated in the CYP2J2-overexpressing mice and was associated with increased plasma levels of 19,20-EDP and 14,15-EET in animals fed the DHA+EPA diet but not the AA-enriched diet. CYP2J2 inhibition in the CYP2J2-overexpressing mice with flunarizine suppressed pathologic choroidal angiogenesis after the laser CNV induction, and cotreatment with montelukast inhibiting CYP2C8 further enhanced the effect. Thus, inhibition of P450s from family 2 was suggested to be a viable approach for suppression of CNV in AMD (Gong et al., 2022).

Attention to the ocular significance of CYP2U1 was brought about by the ocular phenotype of patients with spastic paraplegia 56, an autosomal recessive neurodegeneration characterized by early-onset progressive lower-limb spasticity and weakness due to mutations in *CYP2U1* (Tesson et al., 2012). Pathogenic *CYP2U* mutations were found to cause ocular manifestations with or without neurologic symptoms. The spectrum of these manifestations included macular degeneration associated or not associated with bilateral macular telangiectasia (abnormalities of the macular retinal vasculature), fibrotic CNV, and macular yellowish punctuate deposits in both eyes (Leonardi et al., 2016; El Matri et al., 2021; Zenteno et al., 2022). Therefore, *CYP2U1* was suggested to be included in the panels of genes tested for macular dystrophies, especially in the presence of macular telangiectasia and/or neurologic manifestations (El Matri et al., 2021).

There seem to be only one study on ocular CYP4A significance. In rats, inhibition of CYP4A activity with two different inhibitors, N-hydroxy-N’-(4-butyl-2-methylphenyl) formamidine and dibromododecenyl methylsulfonimide, decreased angiogenic response in the cornea. Accordingly, a CYP4A product, possibly 20-HETE, was suggested to play a critical role in the regulation of corneal angiogenesis and serve as a useful target for reduction of pathologic angiogenesis (Chen et al., 2005).

CYP4B1 was investigated for ocular significance in multiple studies and was shown to mediate hypoxia-induced corneal 12-HETE and 12-HETrE production (Mastyugin et al., 1999, 2001, 2004). Also, all*-trans* and 9-*cis* retinoic acids were found to increase the *CYP4B1* expression and enhance the production of the inflammatory 12-hydroxyeicosanoids in the corneal epithelium (Ashkar et al., 2004). To confirm CYP4B1 involvement in corneal neovascularization, *CYP4B1* was transfected into rabbit cornea in vivo, which led to increased corneal 12-HETrE production and neovascularization (Mezentsev et al., 2005). Conversely, in rabbits, subconjunctival injection of *CYP4B1* siRNA decreased corneal 12-HETrE production and neovascularization (Seta et al., 2007). Collectively, these data suggested CYP4B1 as a component of the inflammatory and neovascular cascade initiated by the corneal injury and that the CYP4B1-12-HETrE system could be a new therapeutic target for preventing corneal neovascularization (Mezentsev et al., 2005; Seta et al., 2007).

Pleiotropic effects and in particular angiogenesis modulation by the P450 metabolites generated from AA, DHA, and EPA provide P450 researchers with multiple golden opportunities. The most obvious is the identification of the enzymes with ocular significance that can serve as pharmacologic targets to contribute to development of new treatments for the most common causes of vision loss. These are retinopathy of prematurity in premature infants, diabetic retinopathy in working-age adults, AMD in the elderly in developed countries, and corneal neovascularization, which affects people of different ages (Gong et al., 2017; Sharif and Sharif, 2019). Yet studies of the individual contributions of the fatty acid-hydroxylating P450s is a challenge, as the five enzymes discussed so far in this section are not the only fatty acid-hydroxylating P450s in mammals (Capdevila et al., 2000; Okita and Okita, 2001; Fer et al., 2008; Arnold et al., 2010; Ni and Liu, 2021). In fact, there are multiple CYP2 and CYP4 isoforms that share extensive amino acid sequence homology, metabolize LPUFAs to similar products, and often have common immunologic determinants. In addition, not all human genes have mouse or rat orthologs (Nelson et al., 2004; Ni and Liu, 2021), making it difficult to study the function of human genes in animals. In addition, many fatty acid-hydroxylating P450s are induced by commonly used drugs, show sex-based differences in expression, and have polymorphic variants (Guengerich, 2005; Jarrar and Lee, 2019; Ni and Liu, 2021), factors that collectively may lead to significant interindividual variability in P450 ocular significance. Nevertheless, all of these challenges could be overcome, at least in part, by careful planning of experiments and more knowledge in the field about the fatty acid-hydroxylating P450s.

CYP4V2 stands apart from the P450s 2C8, 2J2, 2U1, 4A, and 4B1 as mutations in its gene cause Bietti crystalline dystrophy (BCD), a rare autosomal recessive disease (Lee et al., 2001). Currently more than 100 disease-causing mutations in *CYP4V2* have been reported, mostly missense, deletion, insertion, splicing, and nonsense mutations, which are either shown or predicted to lead to the enzyme loss of function (Lee et al., 2001; Nakano et al., 2012; García-García et al., 2019). BCD is manifested by multiple glistening intraretinal crystals (most cases also have similar crystals at the corneal limbus), a characteristic degeneration of the retina, and sclerosis of the choroidal vessels, ultimately leading to progressive night blindness and constriction of the visual field (Li et al., 2004; García-García et al., 2019). The precise chemical composition of the crystals found in patients with BCD is unknown, although studies of human RPE cells generated from patient-induced pluripotent stem cells (iPSCs) provided some insights (Hata et al., 2018). These RPE cells showed the accumulation of glucosylceramide and free cholesterol, and the accumulation of the latter was suggested to cause cell damage and subsequent cell death via the induction of lysosomal dysfunction and impairment of autophagy flux (Hata et al., 2018). In addition, studies of other cell types cultured from patients with BCD (fibroblasts and lymphocytes) demonstrated the absence of two fatty acid-binding proteins, abnormally high triglycerides and cholesterol storage, and reduced conversion of fatty acid precursors into *ω*-3 LPUFAs (Lee et al., 1998, 2001). *Cyp4v3^−/−^* mice (the mouse ortholog of CYP4V2) were generated and found to recapitulate the characteristic features of corneoretinal crystal accumulation and systemic dyslipidemia seen in BCD (Lockhart et al., 2014).

Two treatment approaches for BCD have been investigated so far. In the first, the increased free cholesterol content in the BCD iPSC-RPE cells was reduced by the cyclodextrin or *δ*-tocopherol treatment, which rescued the BCD phenotypes. These data suggested that local cholesterol metabolism may play a role in the pathogenesis of BCD and that decreasing the intracellular free cholesterol content may have therapeutic efficacy in patients with BCD (Hata et al., 2018). In the second approach, multiple cell lines were evaluated for transduction with the *CYP4V2-*containing adeno-associated virus as the clinical characteristics of BCD are believed to be ideal for gene therapy through subretinal injections. The best protein expression and enzyme activity were found with the iPSC-RPE cells and the codon optimized *CYP4V2*, thus supporting the development of *CYP4V2* gene therapy for BCD treatment (Wang et al., 2022).

CYP4V2 is expressed ubiquitously in human tissues, including brain, placenta, lung, liver, and kidney (Li et al., 2004; Nakano et al., 2012). Yet the disease phenotype seems to be restricted to the eye. Thus, some of the opportunities in studies of CYP4V2 are to ascertain the exact molecular mechanism(s) underlying the BCD ocular phenotype and to develop gene therapy or pharmacologic treatments for this currently incurable disease.

#### Vitamin-Metabolizing P450s

In the eye, these P450s are represented by the enzymes (26A1, 26B1, 26C1, and 27C1) that pertain to vitamin A, which is obtained from diet as it cannot be synthesized endogenously (von Lintig et al., 2021). Vitamin A is found at the highest concentrations in the eye (Luo et al., 2006), likely a reflection of its ocular significance. Indeed, vitamin A and its derivatives are essential for eye development as ocular malformations are the most sensitive indicators of fetal vitamin A deficiency and night blindness is an early sign of postnatal deficiency (Wilson et al., 1953). Also, after birth, the vitamin A derivative 11-*cis*-retinal is required for the visual cycle in the retina of humans and many other species and thus is critical for vision (Choi et al., 2021).

Of the ocular vitamin A-metabolizing P450s, CYPs 26A1, 26B1, and 26C1 are believed to be the main hydroxylases and degrading enzymes of all-*trans*-retinoic acid, the biologically active derivative of vitamin A_1_ (all-*trans-*retinol). In addition, CYPs 26A1, 26B1, and 26C1 can hydroxylate 4*(S)*- and 4*(R)*-hydroxy-all-*trans*-retinoic acid, 4-oxo-all-*trans*-retinoic acid, 9-*cis*-retinoic acid, and 13-*cis*-retinoic acid (Isoherranen and Zhong, 2019). CYP27C1, the forth vitamin A-metabolizing P450, was previously classified as an orphan P450 (a P450 whose endogenous or exogenous substates were unknown at the time of gene identification; Guengerich et al., 2005). However, recently it was shown to convert vitamin A_1_ into vitamin A_2_ (3,4-didehydroretinol) in vivo (zebrafish). In vitro, CYP27C1 was found to efficiently metabolize vitamin A_1_, retinal, and retinoic acid and had the highest catalytic efficiency with vitamin A_1_ (Enright et al., 2015).

Studies of vitamin A-metabolizing P450s in the eye are currently very limited and include only a few papers. *Cyp26a1* and *Cyp26c1* were shown to have coordinate expression in mouse retina during eye development; however, the expression of both enzymes became undetectable after postnatal day 14 (Sakai et al., 2004; Luo et al., 2006). In *Xenopus laevis* (the African clawed frog), CYP26A1 and CYP26B1 were found in both normal and regenerating corneas, and the expression of *Cyp26a1*, *Cyp26b1*, and *Cyp26c1* was also detected in the lens, where the CYP26 activity was shown to be necessary for lens regeneration (Thomas and Henry, 2014). The ability of CYP27C1 to generate 3,4-didehydroretinol in zebrafish was discovered to underlie the unusual fish visual sensitivity beyond the range of human vision (a so-called red-shifted photosensitivity). This was because 3,4-didehydroretinol is the precursor of 11-*cis*-3,4-didehydroretinal, the visual chromophore in species with red-shifted photosensitivity as compared with 11-*cis*-retinal, the visual chromophore in humans and species with the range of human vision (Enright et al., 2015). Thus, an opportunity for P450 researchers is to ascertain the role of CYPs 26A1, 26B1, and 26C1 in the cornea and lens as well as to clarify whether these enzymes are important in the retina postnatally. As for CYP27C1, an opportunity is to establish its preferred endogenous substrates in different species and its ocular significance in humans.

#### Eicosanoid-Metabolizing P450 4F8

CYP4F8 was identified as the AA hydroxylase that mostly generates 18-HETE as well as a prominent prostaglandin H_1_ and H_2_ *ω*-2 hydroxylase that mostly yields 19(*R*)-hydroxylated products (Bylund et al., 2000). While originally discovered in the epithelium of human seminal vesicles (Bylund et al., 1999), this P450 was also immunolocalized to the corneal epithelium (Stark et al., 2003). However, the substrate preferences of CYP4F8 in the corneal epithelium and its corneal significance are currently unknown, representing an opportunity for P450 researchers.

## Conclusions and Practical Suggestions

P450 expertise is needed in research efforts to prevent and combat blindness, the most feared condition in the US adult population, and numerous opportunities exist in the P450 research on the eye. Thus, a practical question is “How can eye-related research be initiated in my laboratory?” Multiple approaches are possible. If starting from scratch, first insights into the potential P450 function in the eye could be obtained by immunolocalizations of this P450 within the eye and its specific cell types, preferably in both human and mouse ocular tissues to ascertain any interspecies differences. If systemic or cell-specific P450 knockouts are available, these mutant mice can undergo ocular examination by the methods specific to the eye structure, where this P450 is expressed. Some of the challenges in this approach is that ocular examinations are not trivial and usually require a collaboration with an expert in the field. Specific equipment should also be available, which ranges from simple and relatively inexpensive (e.g., a direct ophthalmoscope for examining the eye fundus) to state-of-the-art and expensive (e.g., an ultra-high resolution spectral domain optical coherence tomograph) to assess retinal gross structure. Fortunately, in many cases, expensive and state-of-the-art ophthalmology equipment is accessible via the National Eye Institute-supported P30 Core facilities, which many universities have to facilitate eye-related research. Finally, it is always a possibility to wait until the ocular significance of a P450 is implicated by genetic and clinical studies or other approaches and then start addressing arising or remaining questions. Regardless of the approach, researchers should educate themselves about different ocular tissues, their structure and function, and initiate a collaboration with a basic scientist or a clinician scientist in the ophthalmology department. In addition to their ophthalmic expertise, these researchers can provide referrals to the private foundations and other organizations that support eye research and thus could be a source of funding. Overall, while challenging, it is possible for P450 investigators to study P450s in the eye, either as an independent research direction or as a collaborative study, as exemplified by the papers cited in this review.

## ABBREVIATIONS

24HC: 24-hydroxycholesterol
AA: arachidonic acid
AMD: age-related macular degeneration
BCD: Bietti crystalline dystrophy
CNV: choroidal neovascularization
CYP: cytochrome P450
DHA: docosahexaenoic acid
EDP: epoxydocosapentaenoic acid
EET: epoxyeicosatrienoic acid
EEQ: epoxyeicosatetraenoic acid
EPA: eicosapentaenoic acid
HETE: hydroxyeicosatetraeonic acid
HETrE: hydroxyeicosatrienoic acid
LPUFA: long-chain polyunsaturated fatty acid
iPSCs: induced pluripotent stem cells
PCG: primary congenital glaucoma
POAG: primary open-angle glaucoma
PPAR: peroxisome proliferator-activated receptor
RPE: retinal pigment epithelium
